# Transactions of the Medical Society of the State of New York

**Published:** 1850-07

**Authors:** 


### ARTICLE V.

*Transactions of the Medical Society of the State of New York, during its Annual Session, held Albany, Feb.* 5, 1850; *pp.* 276.

The first article in this volume is the Annual Address, from the President, Dr. Alex. H. Stevens, of N. Y. city, upon the subject of the "The Public Health." It occupies twenty-seven pages, and is replete with useful information upon the subjects of the importance of State sanitory regulations, ventillating buildings, and drainage.

The second article is a clear and forcible argument, by the same author in favor of the opinion that Asiatic Cholera is communicable.

The third paper is "An Historical Sketch of the state of medicine in the American Colonies, from their first settlement to the period of the Revolution: By Jno. B. Beck, M. D., &c," of which we are also indebted to the able author for a copy of the second edition in a separate form.

The fourth article is made up of "Contributions to the vital statistics of the State of New York; by Lemuel Shattuck, Esq., of Boston."

These are valuable papers, as, indeed, are several others that follow.

But our space is too much crowded to give it a further notice.

Dr. Alexander Thompson, of Cayuga Co., was elected President for the ensuing year.

